# Monolingual and Bilingual Infants’ Ability to Use Non-native Tone for Word Learning Deteriorates by the Second Year After Birth

**DOI:** 10.3389/fpsyg.2018.00117

**Published:** 2018-03-15

**Authors:** Liquan Liu, René Kager

**Affiliations:** ^1^School of Social Sciences and Psychology, Western Sydney University, Sydney, NSW, Australia; ^2^Utrecht Institute of Linguistics-OTS, Utrecht University, Utrecht, Netherlands; ^3^MARCS Institute for Brain, Behaviour & Development, Western Sydney University, Sydney, NSW, Australia; ^4^Centre of Excellence for the Dynamics of Language, Australian Research Council, Canberra, ACT, Australia

**Keywords:** label–object mapping, lexical tone, bilingualism, interpretive narrowing, perceptual assimilation

## Abstract

Previous studies reported a non-native word learning advantage for bilingual infants at around 18 months. We investigated developmental changes in infant interpretation of sounds that aid in object mapping. Dutch monolingual and bilingual (exposed to Dutch and a second non-tone-language) infants’ word learning ability was examined on two novel label–object pairings using syllables differing in Mandarin tones as labels (flat vs. falling). Infants aged 14–15 months, regardless of language backgrounds, were sensitive to violations in the label–objects pairings when lexical tones were switched compared to when they were the same as habituated. Conversely at 17–18 months, neither monolingual nor bilingual infants demonstrated learning. Linking with existing literature, infants’ ability to associate non-native tones with meanings may be related to tonal acoustic properties and/or perceptual assimilation to native prosodic categories. These findings provide new insights into the relation between infant tone perception, learning, and interpretative narrowing from a developmental perspective.

## Introduction

As new language learners, young infants need to determine the possible sound forms in the ambient environment that entail lexical relevance. They must learn to ignore acoustic sound contrasts that do not carry meanings. This task may be more challenging for infants exposed to more than one language, which accounts for more than 50% of the world population (Grosjean, 2010), although how bilingual infants acquire language is largely derived from research studying monolingual infants. Similarities between monolingual and bilingual developmental trajectories can reveal fundamental learning mechanisms and highlight the nature of bilingual learning, whereas differences may reflect specific learning strategies and outcomes stemming from different learning environments. Tone languages consist of more than 60% of the world’s languages (Yip, 2002). The current study adds to our understanding of non-native tone-language learning and investigates the intersection of linguistic and lexical development by examining the learning of minimal pairs involving a tonal contrast across non-tone-language learning monolingual and bilingual infants in the second year after birth.

Infants have an astounding sensitivity to speech sounds in the ambient environment. As such, newborns discriminate between non-native languages through different rhythmic classes (Mehler et al., 1988; Nazzi et al., 1998a), pitch contours (Nazzi et al., 1998b), and lexical stress patterns (Sansavini et al., 1997). In the first year of life, infants tune in to their native sound inventories and tune out of non-native contrasts, a process known as perceptual attunement (Werker and Tees, 1984; Anderson et al., 2003; Kuhl et al., 2006; Watson et al., 2014). The language-specific attunement occurs around 8–12 months for consonants (Werker et al., 1981; Best et al., 1995) and around 6–8 months for vowels (Kuhl et al., 1992). By the end of the first year, infants’ sensitivity to non-native consonant and vowel contrasts greatly decreases. These perceptual patterns extend to adulthood (Tsushima et al., 1994; Tsao et al., 2000). As for the attunement of lexical tones, tone-language learning infants maintain and improve their tonal sensitivity (Harrison, 2000; Mattock and Burnham, 2006; Yeung et al., 2013; Shi et al., 2017a; Tsao, 2017). Meanwhile, non-tone-language learning infants’ sensitivity to tones greatly decreases at 9 months (Mattock and Burnham, 2006; Mattock et al., 2008; Liu and Kager, 2014; Cabrera et al., 2015; Shi et al., 2017b).

After perceptual attunement, listeners do not appear to totally lose sensitivity to tones. Instead, categorical perception (Hallé et al., 2004; Xu et al., 2006; Chen et al., 2015) and neuroimaging studies (Gandour et al., 2000; Kaan et al., 2008) suggest non-tone-language listeners are sensitive to tones, though perceiving them in an acoustic manner. In other words, non-tone-language listeners appear to demonstrate acoustic instead of linguistic processing of tones, determined by a number of factors across contexts, experiences, and modalities (Burnham et al., 2015a,b). A tonal sensitivity rebound has been found in non-tone-language learning infants in the second year after birth, resulting in a U-shaped tonal perceptual trajectory (Liu and Kager, 2014). Tested in a visual habituation paradigm, Dutch monolingual infants show a rebound in sensitivity to a tonal contrast at 17–18 months. No similar U-shaped pattern across the first 2 years after birth has been reported for the perception of non-native consonant and vowel contrasts (Liu and Kager, 2015a,b). However, since non-tone-language adult listeners are sensitive to tones (Xu et al., 2006), such rebound is not entirely unexpected. The rebounded sensitivity is attributed to infants’ acoustic (or phonetic, hereinafter) rather than linguistic (or phonological, hereinafter) sensitivity in light of non-tone-language adult listeners’ acoustic perception of tones (Hallé et al., 2004; Jongman et al., 2017). This is similar to English infants’ discrimination of non-native Zulu click sounds given the acoustic dissimilarity of these sounds to native inventory (Best et al., 1988, 1995).

Question arises whether infants growing up learning two languages follow the same trajectory as monolinguals. Following the previous study reporting U-shaped tonal perceptual trajectory (Liu and Kager, 2014), a follow-up study reports that non-tone-language learning infants from bilingual backgrounds show a rebound to the same tonal contrast at approximately 11–12 months after birth, 6 months earlier than their monolingual peers. Similar findings have been reported for the perception of consonant and vowel contrasts (Bosch and Sebastián-Gallés, 2003; Burns et al., 2007; Albareda-Castellot et al., 2011; Liu and Kager, 2016b). Similar to that of monolinguals, the rebounded sensitivity in non-tone-language learning bilingual infants matches adult data in suggesting that non-tone-language listeners perceive lexical tones acoustically (Hallé et al., 2004; Jongman et al., 2017). More generally, it matches previous literature showing that adult tone-language and non-tone-language listeners use different acoustic cues when perceiving lexical tones (Lee et al., 2008, 2010; Huang and Johnson, 2010; Cabrera et al., 2014).

Additionally, several factors such as enhanced auditory sensitivity and cognitive advantages have been proposed to account for the bilingual perceptual difference (Liu and Weidemann, 2017). The rebounded sensitivity in non-tone-language learning infants and the bilingual difference are crucial for the current study as they lead to further questions: What is the nature of non-tone-language learning infants’ tonal perception at the rebound stage: acoustic or linguistic? Does rebounded sensitivity influence infants’ learning ability of non-native words? Furthermore, does a bilingual difference in perception lead to a better outcome in learning?

These questions can be answered through label–object mapping involving non-native tonal minimal pairs. Specifically, if infants perceive tones linguistically at the rebounded stage, they are expected to be able to learn words contrasting in tone. Alternatively, if their perception is acoustically driven, non-tone-language learning infants’ tonal word learning ability should deteriorate with age. Infants initially accept a wide range of word forms (e.g., non-speech sounds; Woodward and Hoyne, 1999; Hirsh-Pasek et al., 2000; Namy, 2001), and recognize early word forms with increasing linguistic experience (Jusczyk and Hohne, 1997; Tincoff and Jusczyk, 1999; Swingley and Aslin, 2002; Fennell and Werker, 2003; Bergelson and Swingley, 2012, 2013). They are able to recognize frequently used words and map novel labels to novel objects as early as 6 months (Shukla et al., 2011; Bergelson and Swingley, 2012). At 12 months, infants have developed native phonotactics, and continue to show label–object mappings for non-native sound contrasts (Jusczyk and Luce, 1994; MacKenzie et al., 2011, 2012). Dutch infants of 18 months interpret vowel duration as lexically contrasting, but English learners of the same age do not (Dietrich et al., 2007). This is in keeping with the different vowel properties of Dutch and English. By 20 months, their ability to associate non-speech symbols or non-native sounds with objects deteriorates (Namy and Waxman, 1998; Woodward and Hoyne, 1999; May and Werker, 2014; Saffran, 2014; Hay et al., 2015). In the second year after birth, infants appear to have formed sound categories from native language which they adopt to guide the acquisition of words, suggesting the experience of a second attunement (Werker and Tees, 2005). That is, infants refine what they consider to be possible word forms, and their early linguistic learning entails not only language-relevant acoustic cues but also linguistic interpretation at appropriate levels of linguistic analysis.

Tested by a label–object mapping paradigm in which infants are required to map two novel sounds with novel objects, 14- and 20-month-olds successfully associate dissimilar-sounding words with novel objects (*lif-neem*; Stager and Werker, 1997; chook-dal, Bijeljac-Babic et al., 2009). They do not typically succeed in associating minimal pair acoustic features with novel objects (*bih-dih*) possibly limited by their low vocabulary size (Werker et al., 2002) or task difficulty (Yoshida et al., 2009). Nevertheless, they are able to do so when additional information is provided, such as (1) increased referential cues (Fennell and Waxman, 2010), (2) additional object familiarization (Fennell, 2012), (3) enhanced speaker variability (Rost and McMurray, 2009), (4) added social interaction (Mani and Plunkett, 2008), (5) reduced task difficulty (visual choice procedure, Yoshida et al., 2009), and (6) similar lexical contexts (frequently occurring phonemes, Thiessen, 2007). At 17–20 months, infants are able to associate novel objects with minimal pair words (Werker et al., 2002). Their performance is tightly correlated with current and future language comprehension and production skills (Bernhardt et al., 2007). Few studies have directly examined the tonal word learning ability among non-tone-language learning monolingual and bilingual infants. English-learning infants succeed in label–object mapping of monosyllabic words that differ in a tonal contrast in Mandarin Chinese (T2 rising vs. T4 falling) at 14 months but fail at 17 (Burnham et al., 2017) and 19 months (Hay et al., 2015). They also fail to associate objects with Mandarin T1 flat vs. T2 rising contrast at 17 months (Burnham et al., 2017). Taken together, infants’ native word learning ability increases between 14 and 20 months, while their non-native word learning ability decreases (Hay et al., 2012, 2015).

It remains unclear whether infants’ diverse linguistic experience may prolong the developmental trajectory in word development. Some studies show non-prolongation of the developmental trajectory. Monolingual and bilingual infants appear to experience linguistic milestones and developmental trajectories at similar time windows (Swain, 1972; Vihman, 1985; Pearson et al., 1993, 1995; Petitto and Kovelman, 2003; Vihman et al., 2007; Werker and Byers-Heinlein, 2008; Werker et al., 2009; Hoff et al., 2012; Werker, 2013; De Houwer et al., 2014; Singh et al., 2014; Liu et al., 2017), with matched number of lexical concepts (Pearson et al., 1993; Pearson and Fernandez, 1994; Junker and Stockman, 2002; Thordardottir et al., 2006; Hoff et al., 2012; De Houwer et al., 2014; Liu et al., 2017). When appropriate contextual carriers are given (e.g., speaker matching their language environment), monolingual and bilingual infants both learn certain minimal pairs (/bos/-/gos/, Mattock et al., 2010; /k𝜀m/-/g𝜀m/, Fennell and Byers-Heinlein, 2014) at 17 months. On the other hand, prolonged word learning trajectory in bilingual infants have also been reported (Friedrich and Friederici, 2004; Conboy and Mills, 2006; Kaushanskaya and Marian, 2009; Marchman et al., 2010; Byers-Heinlein, 2013; Singh, 2017; Singh et al., 2017). Nine-month-old Chinese–English bilingual infants recognize both Chinese and English words contrasted in tone (Singh and Foong, 2012), even though English does not use tone to differentiate meaning. While monolingual infants begin to succeed at learning two minimal paired non-words (/bI/-/dI/) around 17 months, bilinguals do not succeed until 20 months (Fennell et al., 2007; Werker, 2013). Bilingual children are behind their monolingual peers in vocabulary size when single language, especially the non-dominant language, is compared from 8 months up to 10 years (Bialystok et al., 2010; Gauthier and Genesee, 2011). Eighteen-month-old bilingual but not monolingual infants keep their flexibility for a prolonged period and continue to show the mapping of labels that minimally contrast in pitch contour to novel objects, and the ability deteriorates at 22 months (Graf Estes and Hay, 2015). Both monolingual English-learning and bilingual English–Chinese-learning infants are able to detect tonal substitutions as mispronunciations at 18 months, but monolingual infants are no longer able to do so at 24 months while the bilinguals were still able to do so. Bilingual infants’ performance appears to be language-specific: they can detect tone mispronunciations in Chinese but not English contexts (Singh et al., 2014). In sum, the results of word learning studies with monolinguals and bilinguals suggest that infants attribute linguistic relevance to tones in a language-specific fashion between 18 and 24 months. By the end of the second year, infants’ ability to use lexical tone for word learning is in accordance with their native language and exposure.

Discrepancies between monolingual and bilingual infants’ novel word learning outcomes may be attributed to a number of factors such as different use of learning strategies (Sebastián-Gallés et al., 2012) and task design (Singh et al., 2012). Infants from a multilingual environment do not use mutual exclusivity to the same degree as their monolingual peers when learning words (Byers-Heinlein and Werker, 2009). They are sensitive to environmental differences and contextual cues (Mattock et al., 2010; Fennell and Byers-Heinlein, 2014). In addition, differences in time windows at which non-tone-language learning infants’ tonal label–object mapping ability decreases may be due to different testing paradigms, which may elicit different levels of sensitivity.

Apart from these factors, acoustic properties of tonal contrast may also play a role. A number of studies use T2–T4 (rising–falling) in Mandarin Chinese as the target contrast (Singh and Foong, 2012; Hay et al., 2015), with the pitch directions close to those in the interrogative-narrative intonation patterns in many languages, such as English and Spanish. This potentially introduces an effect of perceptual assimilation, assimilating a non-native phoneme into one’s native phonemic category (Best, 1994; Soderstrom et al., 2011; Tyler et al., 2014). If perceptual assimilation plays a role in tonal processing and learning, we would expect non-native listeners’ better performance in T2–T4 (rising vs. falling) compared to the T1–T4 contrast, in which the assimilation of T1 remains unclear. In addition, an expansion of investigation to other non-native tonal contrasts and re-examination of the word learning of 18-month-old non-tone-language learning infants is necessary to further understand the impact of age, stimuli, and language background on *interpretive narrowing* in word learning (Stager and Werker, 1997; Hay et al., 2015), the process by which infants restrict the types of sounds that can be mapped to word meanings.

To understand the impact of linguistic diversity on novel word learning ability, we tested monolingual and bilingual infants, both of whom lacked prior experience to lexical tones. To reduce the effect of perceptual assimilation on tonal word learning, a new tonal contrast different from previous studies (T2 rising vs. T4 falling) was used. Linking the previous question concerning the nature of tone perception among infants learning non-tone-languages, the research questions are: How does non-tone-language learning Dutch infants’ label–object mapping ability for sound-to-meaning pairs involving lexical tone contrasts develop during the second year of life? Do learning patterns differ between non-tone-language learning monolingual and bilingual Dutch infants? We adopted a word learning paradigm using the same stimuli as in the previous visual habituation paradigm (Stager and Werker, 1997) and tested monolingual and bilingual infants at two age ranges (14–15 and 17–18 months). To understand the effect of contrast acoustic properties on learning and reduce the effect of potential perceptual assimilation, a contrast in Mandarin Chinese (T1 level vs. T4 falling) was used. We predicted successful learning at 14–15 months for both monolingual and bilingual Dutch infants and left the prediction open for 17- to 18-month-olds. We hypothesize that the tonal rebound is acoustic/phonetic in nature and hence would not positively affect word learning. Bilingual infants may show enhanced performance for word learning due to their flexibility in learning non-native tone-to-word pairings (Graf Estes and Hay, 2015). Alternatively, bilingual infants undergo the perceptual rebound earlier than monolinguals. These two factors may affect bilinguals’ ability to learn new words contrasting on tones at 17–18 months.

## Experiment 1

### Participants

Sixty-four (30 male) typically developing Dutch infants aged 14–15 months participated in the experiment. All bilingual infants had a non-tone or pitch-accented language as the other native language since birth apart from Dutch (mean Dutch exposure 55 ± 15%). Evaluated by a multilingual infant questionnaire (Liu and Kager, 2016a), infants’ degree of exposure to the non-dominant language was no less than 20%. Participating families come from similar social economic backgrounds with the same level of parental education. Data from 14 infants were excluded for: fussiness (4), crying (3), and inattentiveness [looking time (LT) less than 1 s in a consecutive of five trials] during the experiment (2), as well as failure in reaching the habituation criterion (5, defined in Procedure). The detailed attrition rate for the individual group is listed in **Appendix 1**. In the final sample, data of 20 monolingual and 20 bilingual infants (bilingual language backgrounds listed in **Appendix 2**) were incorporated into the analysis (mean age: 447 ± 13.7 days). Parents of the participants confirmed no language impairments as well as normal hearing for their children, and provided written informed consent for the study. At present and at the time of the study, the experiment endorses the *WMA Declaration of Helsinki - Ethical Principles for Medical Research Involving Human Subjects*, as well as *The Netherlands Code of Conduct for Scientific Practice* issued in 2004 (revised in 2012) by the Association of Universities in the Netherlands (VSNU). The Ethical Assessment Committee of Utrecht Institute of Linguistics, Utrecht University offered a positive advice on the current study.

### Stimuli

**FIGURE 1:**
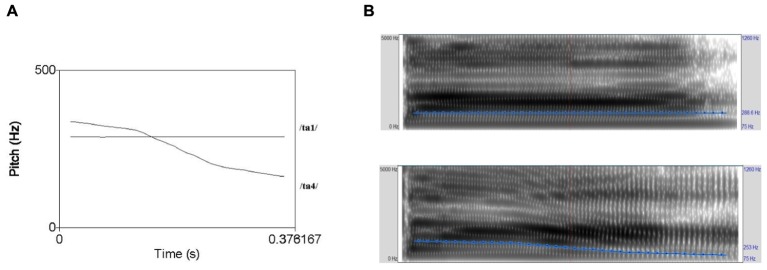
**(A)** Example of fundamental frequency representation of two tokens of the tonal contrast used in Liu and Kager (2014, 2017a,b,c); **(B)** spectrograms of the tonal contrast shown in **Figure 1** (*x*-axis: duration; *y*-axis: amplitude).

**FIGURE 2:**
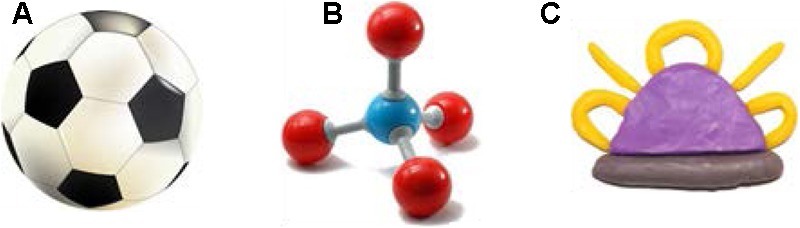
Visual stimuli: **(A)** familiar object in pre-test/ post-test phases; **(B,C)** novel objects in habituation and test phase.

A Mandarin tonal contrast, not tested in previous word learning study (T1 level vs. T4 falling), was selected to create the stimuli for the label–object association in the current study. The syllable /ta/ was selected as the tone-bearing syllable. /ta1/ “build” and /ta4/ “big” are both legal words in Mandarin Chinese. A Mandarin female speaker’s speech production was recorded by Audacity (open source computer program) via Genelec 1029A active speaker recording system in a sound-attenuated room of Utrecht Institute of Linguistics, Utrecht University Phonetics Lab. Four natural pairs of T1–T4 were recorded for each sound to increase within-speaker variation. Examples of pitch contour and spectrograms of a T1–T4 pair of stimuli was provided in **Figures 1A,B**. A ball is selected as the familiar stimulus, and the novel objects consisted of two distinct, multicolored images moving back and forth horizontally on the monitor (**Figure 2**).

### Procedure

A version of label–object mapping paradigm similar to previous studies (Graf Estes and Hay, 2015; Hay et al., 2015) was adopted. The paradigm included a pre-test, a habituation, a test and a post-test phases. In the pre-/post-test phases, infants saw a moving ball along with 10 tokens of the word “ball.” The purpose was to test infants’ initial and general attention, as well as familiarized them with the program. During habituation, infants were familiarized with the associations between two novel moving objects (**Figure 2**) and the corresponding sound labels (**Figure 1**). The novel label–object pairings were counter-balanced across infants, such that some infants were familiarized with Object1–T1 and Object2–T4 pairs, and the others on Object1–T4 and Object2–T1 pairs. Infants went through two to six blocks depending on their speed of habituation. Each block has four trials, two for each label–object mapping. Within each block, the trial orders were quasi-randomized among six non-repeated options: AABB, ABBA, ABAB, BAAB, BABA, and BBAA. The trials were infant-gaze controlled with maximally 20 s per trial. Each trial ended after infants’ looked away for 2 s consecutively. The inter-stimulus interval was 1 s across phases. When participants’ LTs to both label–object pairings dropped to 65% within a block compared to those in the first block, the habituation criterion was reached. Infants failing to reach this criterion within a maximum of six blocks were excluded from analysis. During the test phase, participants had four trials in either Switch–Same–Switch–Same or Same–Switch–Same–Switch orders. In the Same trials, participants heard the same label–object mappings as during habituation. In the Switch trials, labels were linked to the opposite objects shown in habituation, leading to discrepancies in the sound-object mapping, breaking the association. For instance, if an infant was familiarized with the Object1–T1 and Object2–T4 pairs during habituation, the Same trials in test would still be Object1–T1 and Object2–T4, and the Switch trials would be Object1–T4 and Object2–T1. A longer recovery of attention (in LT) during the broken association in comparison to the familiarized mapping would suggest that infants have successfully established the mapping in the habituation phase. Data of two instead of one trial per trial type (Same vs. Switch) were collected to ensure that the results obtained in the test phase were not by random. The test ended with a happy Dutch song “Alle eendjes zwemmen in het water” (“All ducklings are swimming in the water”) to enhance infants’ joyful emotions when leaving the test booth.

In a sound-attenuated test booth of Utrecht Institute of Linguistics, Utrecht University, infants were seated on their caretaker’s lap, facing a flat screen monitor, a hidden loudspeaker and a hidden camera approximately 1 m away. Infants’ responses were observed through a closed circuit TV. An experimenter recorded infants’ LTs using a button box. The test was presented using the *Flexible Experimental Programme* (Veenker, 2007) designed by university technician based on C. Caretakers and experimenters were blind to the audio stimuli by listening to masking music over headphones during the entire test.

### Results

**FIGURE 3:**
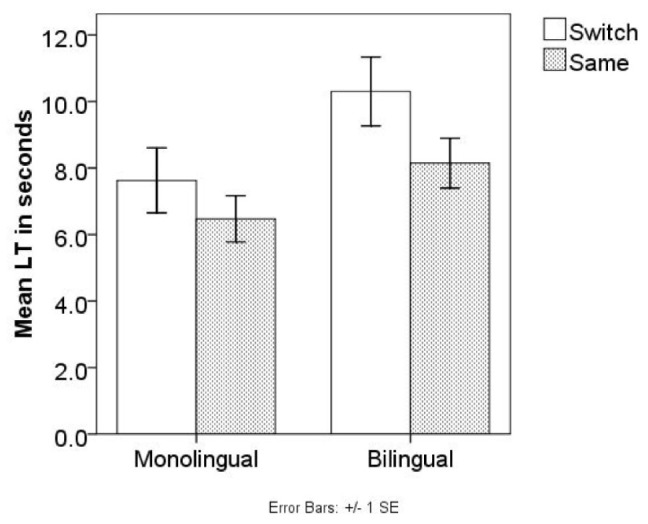
Mean LTs of the Same/Switch trials in Experiment 1 test phase.

A repeated measures analysis of variance (ANOVA) was conducted with the average LT during test as the dependent variable, Trial type (Same vs. Switch) as a within-subject factor, and Language (monolingual vs. bilingual) as a between-subject factor. The effect of Trial type was significant, *F*(1,38) = 8.467, *p* = 0.006, η^2^ = 0.182. The interaction between Language and Trial type was not, *F*(1,38) = 0.161, *p* = 0.691, η^2^ = 0.004. Data suggested that all infants succeeded in labeling a novel non-native tonal contrast with novel objects. Tests of between-subject effect showed that Language was not a significant factor, *F*(1,38) = 0.520, *p* = 0.475, η^2^ = 0.014. Both monolingual and bilingual infants showed longer LT in Switch trials than in Same trials (**Figure 3**) in the test phase. In addition, infants’ habituation time, habituation direction, or the number of blocks did not differ between monolingual and bilingual infants (*p*s > 0.361). Both monolingual and bilingual infants appeared to learn the minimal pairs contrasted in tones. To further investigate non-tone-language learning infants’ word learning ability, we tested infants of an older age in the next experiment.

## Experiment 2

### Participants

Fifty-one (25 male) typically developing Dutch infants of 17–18 months participated in the study. The same language background criteria as in Experiment 1 were adopted. Data from the 11 infants were excluded for: fussiness (2), crying (3), and inattentiveness (1), failure in reaching the habituation criterion (4), and dyslexic background in the family (1). In the final sample, data of 20 monolingual and 20 bilingual infants (language background listed in **Appendix 2**) were incorporated into the analysis (mean age: 537 ± 12.3 days).

### Stimuli and Procedure

The same stimuli and Procedure as in Experiment 1 were adopted.

### Results

**FIGURE 4:**
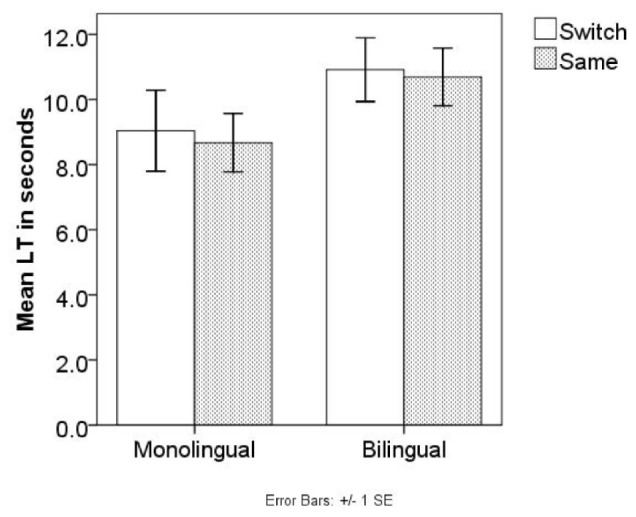
Mean LTs of the Same/Switch trials in Experiment 2 test phase.

A repeated measures ANOVA was conducted with the same factors as in Experiment 1. The main effect of LT between Same and Switch was not significant, *F*(1,38) = 1.642, *p* = 0.208, η^2^ = 0.041, nor was the interaction between Language and Trial type, *F*(1,38) = 0.001, *p* = 0.976, η^2^ < 0.001. Tests of between-subject effect shows that the effect of Language was not significant, *F*(1,38) = 0.009, *p* = 0.925, η^2^ < 0.001. Neither monolingual nor bilingual infants showed longer LT in Switch (**Figure 4**). In addition, infants’ habituation time, habituation direction, or number of blocks did not differ between monolingual and bilingual infants (*p*s > 0.400).

## Discussion

This paper investigated the ability to learn label–object associations of a non-native tonal contrast in toddlers acquiring non-tonal languages, testing the generality of the interpretive narrowing. A tonal contrast different from previous studies (Singh et al., 2014; Graf Estes and Hay, 2015) was adopted for a better understanding of the effect of acoustic properties and perceptual assimilation on learning. Results shed light on Dutch infants’ non-native interpretive narrowing process with two main findings. First, infants were able to establish associations between novel tones and objects at 14–15 months, whereas they failed to do so at 17–18 months. Second, the current results indicated similar developmental trajectories between monolingual and bilingual infants in word learning involving novel and non-native sound contrasts.

### Infant’s Fast Label–Object Mapping of Non-native Tones

Infants maintain detailed representations from the input, paying attention to acoustic, linguistic, and many other cues (Swingley and Aslin, 2002). Nevertheless, they need to ignore variabilities from the input in order to form abstract categories. Between learning stages of sounds and words, an interpretive narrowing in infants’ usage of acoustic detail has been suggested (Stager and Werker, 1997). The finding that 14- to 15-month-old non-tone-language learning infants were able to establish associations between novel tones and novel objects is in line with previous studies, indicating that pitch contour may remain an important acoustic cue for word learning. Infants of 17–18 months, however, no longer exhibit a learning effect, showing incongruent results (Graf Estes and Hay, 2015; Hay et al., 2015). The overall trend suggests a reduction of linguistic function in non-native tones among non-tone-language learning infants, conforming with trends to the interpretive narrowing of consonant or vowel contrasts.

The observed decrease may be attributed to a natural decay of linguistic function with no relevant exposure from the environment under a second perceptual attunement (Werker and Tees, 2005) where infants concentrate on selecting the lexically contrastive properties from their native language. Contrasts that are not relevant to infants’ native language may remain acoustically perceptible (Best et al., 1988). Nevertheless, they are not used for a linguistic function. Since no systematic functional use of lexical tones is present, non-tone-language learning infants never develop tonal categories to map the relevant input. Nor do they pay attention to the tonal variation on a lexical level, exemplifying a “use it or lose it” scenario. It is not a decreased tonal sensitivity that affects the ability to abstract and form categories because of the sensitivity rebound observed in previous studies (Liu and Kager, 2014, 2017c). The deterioration may also reflect the loss of a general ability to abstract as well as create a tonal proto-category. Establishing a lexical representation requires building a link between acoustic exemplars from the ambient environment and word meaning, and subsequently setting up an abstract, categorical representation. After (the first) perceptual attunement, infants have established category boundaries based on their native language inventories and set up categories that matter in meaning differences to guide word learning. It thus becomes increasingly difficult to create new representations for non-native input. Non-tone-language learning infants’ decreased tonal sensitivity may affect their ability to abstract and form categories for unattended acoustic dimensions. This is similar to studies discussing (late) learners’ relative difficulties with specific non-native words (Best and Tyler, 2007; Best et al., 2009). However, this explanation does not conform to infants’ rebounded tonal sensitivity at 17–18 months reported in previous studies (Liu and Kager, 2014, 2017b), which should facilitate generalization of tonal categories.

Linking perception with label–object mapping, successful learning involving a non-native contrast may rely on a number of elements including the exposure to that contrast (Kaan et al., 2007; Liu and Kager, 2011, 2017c), the residual ability of creating categories from acoustic input, and the potential interference from native categories.

### The Effect of Bilingualism on Infant Language Development

The current experiment does not find any significant differences between monolingual and bilingual word learning abilities. Although native sound and word learning trajectories remain debatable between monolingual and bilingual infants, similar learning patterns were found in the current study. This pattern is similar to some word learning experiments showing that early bilingual exposure does not interfere with infants’ fundamental word learning ability (Mattock et al., 2010; Byers-Heinlein et al., 2013), but different from some other experiments in which advantages (Singh et al., 2014; Graf Estes and Hay, 2015; Burnham et al., 2017) or delays (Fennell et al., 2007) are observed in bilingual population. Without relevant input to establish sound categories, neither monolingual nor bilingual non-tone-language learning infants appear to treat word-level pitch as linguistically relevant in the second half of the second year after birth.

### Toward an Integrative View of Non-native Tonal Word Learning

The same stimuli were used in our previous studies in which a visual habituation paradigm was adopted to track non-tone-language learning monolingual and bilingual infants’ discrimination from 5 to 18 months (Liu and Kager, 2014, 2017b). The earlier results in relation to the current ones need to be discussed in order to compare the development of tonal discrimination and word learning ability. Non-tone-language learning infants discriminated the same tone contrast at 14–15 and 17–18 months. Lexical representations may be encoded in fine details, even though these details may not be necessary for linguistic functions such as native vocabulary acquisition (Swingley and Aslin, 2002). Although infants’ auditory sensitivity to non-native tones is rebounded in later infancy and presumably extends to adulthood, 17- to 18-month-old infants do not show label–object mapping using non-native lexical tones in the current study. We hypothesize that non-tone-learning infants show an acoustic instead of linguistic perception of tones by the end of 2 years after birth, resembling non-tone-language adults (Hallé et al., 2004; Jongman et al., 2017).

Data from the current experiments are crucial to the understanding of the time-course of infant word learning ability under study. In line with previous studies (Graf Estes and Hay, 2015; Hay et al., 2015), infants map non-native tonal contrasts to novel objects at 14–15 months, suggesting flexibility in word learning ability for non-native contrasts even after tonal perceptual attunement (Werker and Tees, 2005). The lack of ability to establish label–object association at 17–18 months, for both monolinguals and bilinguals, is consistent with previous findings of monolingual infants (Hay et al., 2015) but contrasting those of bilinguals (observed at 22 months, Singh et al., 2014; Graf Estes and Hay, 2015). Such difference may be attributed to a number of factors such as stimuli or testing paradigms. The procedure used in Singh et al. (2014) introduces two phases of familiarization before training infants on novel label–object mappings: first, participants are familiarized with the task procedure using frequent word–object pairs, and secondly, novel objects are directly presented to the infants. This practice may largely reduce the task difficulty and lead to a better learning effect, resulting in successful mapping at a relatively later age. Moreover, the difference across studies may also be due to an effect of perceptual assimilation of the non-native contrasts (e.g., successful learning of the T2–T4 contrast in Graf Estes and Hay (2015) vs. unsuccessful learning of T1–T4 in the present study). The distinction between T2 and T4 (rising vs. falling) may be better assimilated and more easily perceived than that between T1 and T4 (flat vs. falling). Although non-tone-language learning infants’ perception is arguably acoustically rather than linguistically based after perceptual attunement, their word learning ability appears to be contrast-dependent, influenced by listeners’ linguistic experience and possibly native categories. Perceptual salience is another factor that may play a role in tone perception. It could be that the T2–T4 contrast tested in previous studies may be more salient than the current T1–T4 contrast. However, English infants of 18 months fail to learn a salient, non-native minimal pair contrasted in vowel duration (Dietrich et al., 2007), indicating that perceptual salience may contribute more to acoustic discrimination (e.g., Best et al., 1988; Liu and Kager, 2014; Ramachers et al., 2017) than to linguistic interpretation and as such its effect may be limited during interpretative narrowing.

By the end of the second year of life, infants may maintain detailed representations of acoustic details supported by their auditory sensitivity, but this sensitivity may not present itself in a label–object mapping task especially given isolated stimuli (Fennell and Waxman, 2010). Infants may retain detailed acoustic information provided their general auditory sensitivity. However, they may focus on establishing abstract categories during category learning. This hypothesis fits the developmental framework of the Processing Rich Information from Multidimensional Interactive Representations (PRIMIR) model (Werker and Curtin, 2005). PRIMIR assumes the availability of rich information in the speech input and proposes infants’ information perception and acquisition along three interactive, multidimensional planes: a general perceptual plane, (meaningful) word form plane, a phonemic plane. In any situation, the processing of input information depends on the joint activity of three dynamic filters: initial perceptual biases, developmental stage, and environmental demands. In the current experiment, for instance, infants’ lexical use of non-native tonal information decreases albeit their initial perceptual biases of the lexical pitch. Their performance in the word learning task is hypothesized to be influenced by the task design as well as the specific tonal contrast acoustics.

Regarding bilingual infants, the PRIMIR model has been further extended to bilingual infants (Curtin et al., 2011). Bilingual infants are required to determine which language is relevant in the context of the specific task at hand (Mattock et al., 2010). As lexical tonal information is absent in the linguistic context of Dutch monolingual and bilingual infants, no difference is observed in the current word learning task. Future models of speech processing may extend their predictions on contrast learning and learnability.

## Conclusion

This paper addresses how early language learners determine which acoustic dimensions in their environment differentiate word meanings. Non-tone-language learning monolingual and bilingual infants are able to construct linguistic representations of Mandarin T1–T4 tones at 14–15 but not at 17–18 months. Linking the current findings with previous literature, we hypothesize that infants’ perception of non-native tones is more acoustic than linguistic in the later phase of language development, that is, mainly based on the acoustic properties of tones. In addition, provided the different outcomes across contrasts (T1–T4 vs. T2–T4) between the current and previous studies, we are inclined to suggest a role for perceptual assimilation in non-native word learning (Best, 1994). That is, non-tone-language learning infants’ tonal label–object mapping ability is affected by intonation contours from their native language, and facilitation may occur when acoustic similarities/overlaps occur between non-native tones and native intonation (e.g., T2–T4). Given that differences may also lie in the paradigms used across associative word learning studies, the suggestion should be considered with caution. Last but not least, bilingual infants appear to at least keep the same pace as their monolingual peers along the word learning of non-native tonal contrasts.

## Author Contributions

LL contributed to the experiments. LL and RK contributed to the manuscript.

## Conflict of Interest Statement

The authors declare that the research was conducted in the absence of any commercial or financial relationships that could be construed as a potential conflict of interest.

## Appendix

**Appendix 1:** Attritions across ages and language backgrounds.

Criteria	14–15 months		17–18 months	
	Monolingual	Bilingual	Monolingual	Bilingual
Fussy	2	2	1	1
Crying	1	2	2	1
Inattentive	2	0	1	0
Unhabituated	2	3	1	3
Dyslexic	0	0	1	0

**Appendix 2:** Bilingual language background.

Second L1	Age groups	
	14–15 months	17–18 months
Czech	1	1
English	5	6
French	0	1
German	5	3
Hebrew	0	1
Italian	1	3
Portuguese	1	0
Spanish	4	3
